# Factors Affecting Compression of the Left Subclavian Artery Bridging Stent In Zone 2 Fenestrated Endovascular Arch Repair

**DOI:** 10.1177/15266028241267753

**Published:** 2024-08-04

**Authors:** Petroula Nana, Antonino Giordano, Giuseppe Panuccio, José I. Torrealba, Fiona Rohlffs, Tilo Kölbel

**Affiliations:** 1 German Aortic Center, Department of Vascular Medicine, University Heart and Vascular Center UKE Hamburg, Hamburg, Germany

**Keywords:** left subclavian artery, fenestrated thoracic endovascular repair, stent, compression, anatomy

## Abstract

**Introduction:**

Left subclavian artery (LSA) preservation during thoracic endovascular aortic repair (TEVAR) has been related to low morbidity. This study investigated the incidence of LSA stent compression in patients managed with fenestrated endovascular arch repair (f-Arch) and evaluated the impact of anatomic and technical factors on LSA stent outcomes.

**Methods:**

A single-center retrospective analysis of patients managed with single-fenestration devices (Cook Medical, Bloomington, IN, USA) for LSA preservation, between January 1, 2012 and November 30, 2023, was conducted. Anatomic (arch type, bovine arch, distance between the LSA and most proximal bone structure, left common carotid artery and aortic lesion, take-off angle, diameter, thrombus, calcification, dissection, tortuosity) and technical parameters (stent type, diameter, length, relining, post-dilation) were evaluated. Stent compression was any ≥50% stenosis (using center luminal line) of the stent compared with its initial diameter. Clinical outcomes included stroke and upper limb ischemia at 30 days and follow-up. Technical outcomes included stent compression and need for reintervention.

**Results:**

Fifty-four cases were included. Only balloon-expandable covered stents were used, and relining during the index procedure was performed in 18%. No stroke or arm ischemia was recorded. One stent compression was detected at 30 days. During follow-up, no stroke or arm ischemia was diagnosed. Nine cases (18%) presented stent compression, with a mean time of stent-compression diagnosis at 18 months (interquartile range [IQR]=37, range=1–58 months) after the index procedure. Five (56%) underwent secondary relining. Follow-up after reintervention was uneventful. Lower distance to the nearest bone structure (compression group [CG]: 11.7±8.9 mm vs non-compression group [NCG]: 23.0±7.8 mm, p=0.003) and higher tortuosity index (CG: 1.3±0.4 vs NCG: 1.2±0.1, p=0.03) were associated with LSA stent compression.

**Conclusion:**

LSA stent compression in patients managed with f-Arch affected 1 in 5 cases, without clinical consequences. Distance to the nearest bone structure and higher tortuosity were associated with LSA stent compression.

**Clinical Impact:**

Fenestrated endovascular arch repair for the preservation of the left subclavian artery (LSA) in patients needing landing within the aortic arch has been performed with encouraging outcomes. This analysis showed that LSA stent compression is met in 18% of patients, without though any clinical consequence. Pre-operative anatomic parameters, as lower distance to the nearest bone structure and higher tortuosity index affect negatively LSA stent performance while stent parameters seem to have no impact.

## Introduction

Left subclavian artery (LSA) preservation during thoracic endovascular repair in patients requiring landing proximal to zone 2 has been related to lower stroke, spinal cord ischemia, and arm malperfusion rates compared with LSA occlusion and current recommendations suggest LSA preservation, even in cases of ruptured thoracic aneurysm.^1–4^ Various approaches have been described to preserve or revascularize the LSA, including surgical debranching with left common carotid artery (LCCA)-LSA bypass or transposition, in-situ fenestration with laser or mechanical means, the parallel graft technique, as well as custom-made devices (CMDs).^5–8^

Fenestrated endovascular arch repair (f-Arch) enables LSA preservation in patients presenting with inadequate landing in zone 3.^5,9^ Comparative studies of f-Arch and thoracic endovascular aortic repair (TEVAR) with LSA debranching showed no significant difference between the techniques, with 100% patency and 20% need for unplanned reintervention rates within the midterm follow-up.^5,10^ Despite that most reinterventions were related to distal thoracic extensions, LSA patency after f-Arch may be affected by stent compression, due to both anatomic and technical factors.^10^ However, data on LSA stent outcomes and their clinical consequences, as the evolution of stroke and arm ischemia, are limited.

This study aimed to investigate the incidence of LSA stent compression in patients managed with f-Arch and evaluate the impact of anatomic and technical factors on LSA compression outcomes.

## Methods

### Patients’ Cohort

The STrengthening the Reporting of OBservational studies in Epidemiology (STROBE) statement was followed.^11^A retrospective single-center analysis of patients managed with a single-fenestration device for the preservation of the LSA was conducted. All devices were preloaded, and CMD, relied on the Zenith platform (Cook Medical, Bloomington, IN, USA). In all cases, a combination of a scallop for the preservation of the LCCA and/or innominate artery (IA; in cases of bovine arch), in addition to a fenestration for the preservation of the LSA was used. Data from patients treated between January 1, 2012 and November 30, 2023, with special focus on the anatomy and bridging stent of the LSA, were recorded and analyzed.

All endovascular aortic interventions were performed in a hybrid operating room with fixed imaging system. Since 2015, fluoroscopic fusion guidance has been used (Vessel Navigator, Phillips Healthcare, Best; The Netherlands). This study complied with the Declaration of Helsinki and according to current state laws, no approval was required from the local ethics committee due to its retrospective nature and unidentifiable information.

#### Inclusion and exclusion criteria

Patients presenting with aortic pathologies involving the aortic arch, requiring either elective or urgent repair, using an f-Arch device for the preservation of the LSA were considered eligible and included in this analysis. A variety of aortic pathologies were addressed, including degenerative or post-dissection aneurysms involving the aortic arch, complicated type B dissections, penetrating aortic ulcers (PAUs), and pseudoaneurysms. None of the patients presented with adequate proximal landing zone distal to the LSA (zone 3).

Cases managed with the same device configuration, but aimed for the preservation of the LCCA were excluded (14 patients), due to the longer proximal extension of the disease and the differences in complexity and clinical outcomes. All patients treated with open repair, surgeon-modified endografts, parallel graft technique, or patients managed with LCCA-LSA bypass and standard TEVAR were excluded from this analysis. In addition, all patients had at least 1 post-operative computed tomography angiography (CTA) scan; regardless the time of CTA performance. Patients with no CTA scan post-operatively were excluded from the analysis.

#### Technical details

Systemic heparinization with a target activated clotting time (ACT) range between 250 and 350 seconds was obtained after arterial access. Details on endograft configuration and deployment have been published previously.^12^ In all cases, the LSA fenestration was pre-catheterized, and a through-and-through hydrophilic 0.035″ 4 m wire was obtained to provide appropriate device orientation and easier catheterization. In all patients, a left brachial artery (LBA) access was used for the advancement of the bridging stent. Balloon-expandable stents are preferentially used to bridge the LSA.^12^ Relining using bare metal stents was at the discretion of the operator and performed in accordance with the intra-operative imaging findings.

Patients undergo an at least 24-hour intensive care unit (ICU) surveillance before being transferred to the ward. Follow-up included CTA before discharge, at 12 months, and yearly thereafter. In case of LSA stent compression ≥ 50% detected during follow-up, an early reintervention was suggested, as a measure of prevention of adverse events. If the patient denied reintervention, a conservative management and close follow-up was recommended. Symptomatic cases and LSA stent compression ≥70% were considered as stronger indications for intervention and a reintervention was recommended in these cases.

#### Data collection

**Figure 1.**
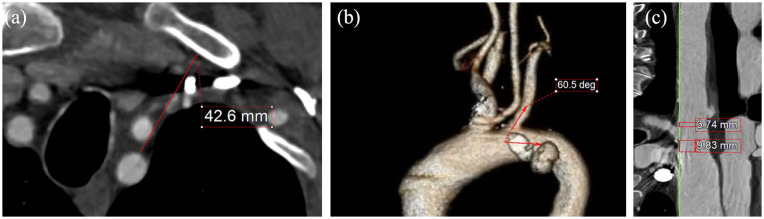
Panel a depicts the way that the minimum distance between the LSA and most proximal bone structure (manubrium of the sternum, clavicula, or first rib was estimated [red line]), while panel b shows the estimation of the take-off angle of the LSA, according to the parallel axis of the aortic lumen (red arrows). The distance between the LSA and the left common carotid artery, as well as the lesion was estimated using outer curve lumen line (panel c).

Age, sex, underlying aortic disease (aneurysm; degenerative or dissective, acute dissection, PAU, pseudoaneurysm) and indication to treat, setting, and maximum aortic diameter were recorded. Regarding anatomic parameters, the Madhwal, et al^13^ classification was used to define aortic arch type (I, II, and III). The presence of bovine arch (common orifice for the IA and LCCA), the minimum distance between the LSA at the expected landing zone of the corresponding bridging stent and the nearest bone structure (manubrium of the sternum, clavicula or first rib, Figure 1a), the take-off angle of the LSA (Figure 1b), the distance between the LCCA and LSA, and LSA and the aortic lesion (both assessed after adjustment of the center line to the outer curve aorta, Figure 1c), the maximum diameter at the expected distal LSA landing zone, and the difference in diameter (Δd) between the ostium and the expected distal LSA landing zone, the presence of thrombus, calcification and dissection 50% within the LSA, and the tortuosity index of the LSA within the landing zone were collected and analyzed.

Technical parameters, including technical success, stent type (balloon vs self-expandable and covered vs bare metal), diameter and length, use of relining with self-expanding bare metal stents, post-dilation, and balloon diameter were recorded. Flaring was performed in all cases. Early and follow-up mortality and stroke, minor or major, events were recorded. Stent-related outcomes during follow-up, including stent fracture or compression, the time of diagnosis, need and type for reintervention, and secondary patency, were assessed.

#### Definitions

**Figure 2.**
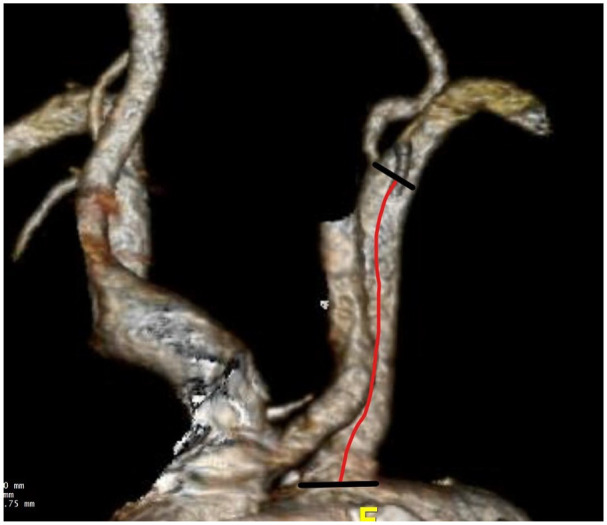
The landing zone of the LSA was defined as the length of the artery extending from the aortic orifice of the LSA up to the most proximal limit of the vertebral artery ostium (red line).

**Figure 3.**
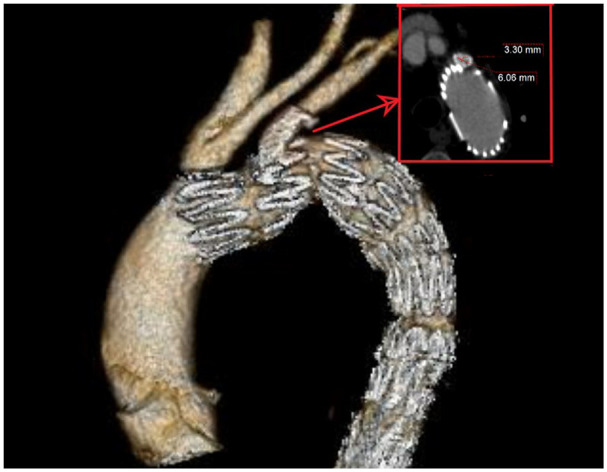
Stent compression was reported as the ≥50% stenosis (minimum diameter in axial view after the application of center lumen line) of the stent compared with its initial diameter, including total stent occlusion. Despite that in 3D reconstruction no stenosis is easily detected the axial view of the stent shows a stenosis >50%, compared with the initial diameter.

**Figure 4.**
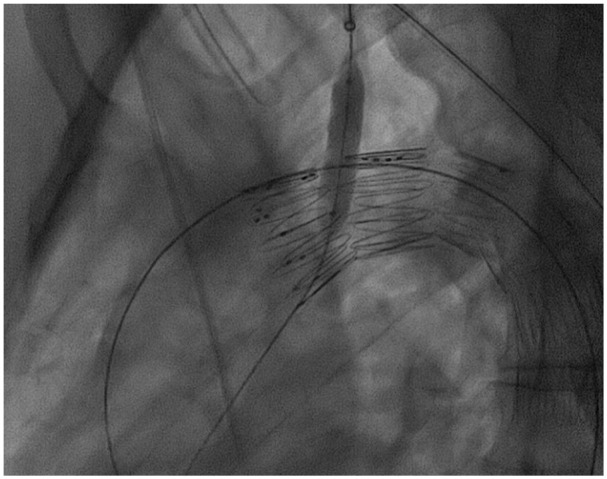
Post-dilation was considered the remodeling of the chosen bridging stent, using a larger diameter angioplasty balloon, to achieve adequate distal apposition.

The expected landing zone of the LSA was defined the length of the artery extending from the aortic orifice of the LSA up to the proximal limit of the vertebral artery ostium (Figure 2). Stent compression was reported as ≥50% stenosis (minimum perpendicular diameter using a center luminal line) of the stent compared with its diameter at the pre-discharge CTA, including total stent occlusion (Figure 3). Technical success was defined as the successful deployment of the aortic endograft and bridging stent deployment into the LSA, without any stent or endograft migration, no LSA occlusion at completion angiography, and survival at least 24 hours after the procedure. Severe calcification or thrombus was reported as the presence of >50% circumferential calcification or thrombus within the landing zone of the LSA.^14^ The tortuosity index of the LSA was automatically assessed. The distance between the LCCA and LSA was calculated using the outer curve lumen line and was defined as the distance from the distal edge of the LCCA ostium to the proximal end of the LSA ostium. To calculate the distance from the LSA to the lesion, the same method was applied, and the distance was defined as the length between the distal end of the LSA to the most proximal limit of the lesion. All measurements were performed by 2 experienced vascular surgeons (P.N. and A.G.) using a dedicated reconstruction software (Terarecon Aquarius iNtuition, v.4.7.2.10-12., San Mateo, CA, USA). For the first 10 cases, the measurements were blindly performed from each researcher. The findings were evaluated by the senior author (T.K.) and no difference over 1 mm was detected at any point. Post-dilation was considered the remodeling of the chosen bridging stent, using a larger diameter angioplasty balloon, to achieve adequate distal apposition (Figure 4). Flaring was performed in all cases at the proximal end of the bridging stent.

#### Outcomes

Clinical outcomes included stroke and upper limb ischemia rates at 30 days and during follow-up. Technical outcomes included stent patency and need for reintervention. The impact of the baseline anatomic and technical factors on LSA bridging stent compression was assessed.

#### Statistical analysis

Normally distributed continuous data were reported as mean±standard deviation and non-normally distributed as median values with range and interquartile range (IQR). Categorical data were expressed as absolute numbers and percentages. Chi-square test was used for categorical data comparison. Independent 2-sample *t*-tests were used for normally distributed continuous variables, and the Mann-Whitney *U*-test for non-normally distributed continuous and ordinal variables; p-value was considered significant when it was <0.05. Statistical analysis was performed by SPSS 29.0 for Windows software (IBM Corp, Armonk, NY, USA).

## Results

During the study period, 54 cases were managed with f-Arch for the preservation of the LSA. Four cases were excluded from this analysis due to lacking follow-up CTA. The mean age was 71.2±5.8 years. Regarding underlying pathologies, 10 patients (20%) were managed for aortic dissection while 24 patients (48%) were treated for degenerative aneurysms, 8 (16%) for PAUs and 8 (16%) for pseudoaneurysms. Among patients, 14% had a history of previous sternotomy.

In terms of baseline anatomic characteristics, 42% of patients had a type II arch, 22% presented a bovine arch, whereas in 2 patients, an aberrant right subclavian artery was present (Table 1). In both cases, a right carotid-subclavian bypass was performed. The mean distance between the LSA landing zone and nearest bone structure was 20.9±9.2 mm while the mean distance between the LCCA and LSA was 12.2±5.0 mm. The mean take-off angle of the LSA was 60±15° and the mean tortuosity index of the LSA was 1.2±0.1. All pre-operative LSA anatomic characteristics are presented in Table 1.

**Table 1.** The Anatomic Characteristics of the Aortic Arch and Left Subclavian Artery (LSA) in Patients Managed with Fenestrated Thoracic Endovascular Repair for the Preservation of the LSA.

Variable, n/N (%) or mean±SD	Total (N=50 cases/LSAs)
*Anatomic characteristics*	
Arch type	
I	8 (16%)
II	21 (42%)
III	21 (42%)
Bovine arch	11 (22%)
LSA characteristics	
Distance from bone structure	20.9±9.2 mm
Distance between LCCA-LSA	12.2±5.0 mm
Distance between LSA**-**lesion	8.8±6.3 mm
Take-off angle	59.8±14.6°
Diameter at landing zone	9.7±1.2 mm
Δ diameter between orifice and landing zone	3.5±1.4 mm
Tortuosity index	1.2±0.1 mm
Calcification	
Severe calcification (>50% of the circumference)	0 (0%)
Moderate calcification (25%–50% of the circumference)	4 (8%)
Mild calcification (<25% of the circumference)	1 (2%)
Thrombus	
Severe thrombus (>50% of the circumference)	0 (0%)
Moderate thrombus (25%–50% of the circumference)	2 (4%)
Mild thrombus (<25% of the circumference)	0 (0%)
Dissection	0 (0%)

LCCA: left common carotid artery; LSA: left subclavian artery.

Regarding technical parameters, only balloon-expandable covered stents were used, while relining with self-expanding bare metal stents was performed in 9 cases (18%). The median stent diameter was 10 mm (IQR=2, range=8–10) and post-dilation was performed in 78% of stents with a median balloon diameter of 12 mm (IQR=0, range=8–14). Details on stent characteristics are presented in Table 2.

**Table 2.** Bridging Stent Characteristics for the Left Subclavian Artery (LSA) in Patients Managed with Fenestrated Thoracic Endovascular Repair.

Variable, n/N (%) or mean±SD	Total (N=50 cases/LSAs)
*Stent characteristics*	
Type of stent	
Balloon-expandable covered stents	50 (100%)
Stent diameter	10 (IQR=2, range=8–16)
Advanta V12 (Atrium Medical Corporation, NH)	47 (94%)
Lifestream (Bard Medical, GE, USA)	2 (4%)
BeGraft Peripheral (Bentley, Innomed, DE)	21 (42%)
Relining with self-expanding bare metal stent	9 (18%)
-Protégé (Medtronic, CA USA)	9 (18%)
Post-dilation	39 (78%)
Balloon diameter	12 mm (IQR=0, range=8–14)
Flaring	50 (100%)

LSA: left subclavian artery.

## Clinical Outcomes

All patients completed the 30-day follow-up. The early mortality rate was 6% (3 patients) while none had a stroke or transient ischemic attack and no patient reported symptoms of arm malperfusion within 30 days. Follow-up was 12 (range=1–84, IQR=23) months with an estimated loss to follow-up at 35%. No stroke or arm malperfusion was detected during follow-up.

## Technical Outcomes

During the 30-day follow-up, 1 LSA stent compression was diagnosed (Figure 3) while no stent fracture or reintervention was reported. The patient with the LSA stent compression was managed after 30 days (2-month follow-up), with relining.

During follow-up, 9 cases (18%) of LSA stent compression were recorded. None had a stent occlusion or clinical consequences. The mean stent diameter after compression was 4 mm (range=3–6 mm) and the mean time of stent-compression diagnosis was 18 months (IQR=37, range=1–58 months). The details on patients presenting with LSA stent compression are presented in Table 3. Reinterventions were performed in 5 (56%) of 9 cases while the remaining 4 patients were managed conservatively with imaging surveillance. In all patients needing reintervention, relining was performed with various stents. The mean time of reintervention was 24 months (2–60 months). The follow-up after relining was 1 month (1–12 months). None of the patients underwent reintervention or presented imaging or clinical signs of recurrence.

**Table 3.** Patients’ Characteristics Diagnosed With LSA Bridging Stent Compression After Fenestrated Thoracic Aortic Aneurysm Repair. Five of the 9 Patients Underwent Reintervention. No Recurrent Stenosis Event was Detected.

Age	Sex	Indication for repair	Arch characteristics	Distance from bone structure	Take-off angle	Diameter	Tortuosity index	Bridging stent	Relining	Balloon for post-dilation	Time of stent-compression diagnosis in months	Reintervention	Time of reintervention in months
74	M	Degenerative thoracic aortic aneurysm	Type II	12	63	9	1.9	Advanta 8/38 mm		10 mm	18		
63	F	Chronic aortic dissection	Type I	17	115	9.5	1.2	Advanta 8/38 mm		10 mm	58	Palmaz 8/18 mm and Protégé Everflex 10/40 mm	12
71	M	Degenerative thoracic aortic aneurysm	Type I	20	39	10	1.2	Advanta 8/38 mm		10 mm	48		
66	M	Degenerative thoracic aortic aneurysm	Type II	8	40	11	1.17	Advanta 10/38 mm			12		
73	M	Degenerative thoracic aortic aneurysm	Type III and bovine	10	30	7	1.1	Advanta 10/38 mm	Protégé Everflex, 12/40 mm	8 mm	48	iCover 10/27 mm	48
76	F	Degenerative thoracic aortic aneurysm	Type III	2	46	6.2	1.42	Advanta 9/38 mm			10		
68	M	Chronic aortic dissection	Type III and bovine	17	35	8	1.3	Advanta 10/38 mm		12 mm	24	Visipro 10/27 mm	24
77	M	Degenerative thoracic aortic aneurysm	Type I	13	45	11	1.25	Advanta 10/38 mm		14 mm	18	VBX 8/59 mm	24
82	F	Degenerative thoracic aortic aneurysm	Type III	6	55	9.8	1.1	Advanta 10/38 mm		12 mm	1	Begraft 6/38 mm and Visipro 7/27 mm	2

## Factors Associated With LSA Stent Compression

Pre-operative anatomic characteristics and technical details were analyzed between patients with or without LSA stent compression. Follow-up within the compression group (CG) was estimated at 40 months vs 15 months within the non-compression group (NCG, p=0.02). The presence of degenerative aneurysm seemed to affect LSA stent compression (p=0.04). In addition, the lower distance from the nearest bone structure (p=0.003) and the higher tortuosity index (p=0.03) affected LSA stent compression (Table 4). Stent parameters, including type, diameter, length, and relining did not affect LSA stent outcomes, as presented in Table 4.

**Table 4.** Anatomic and Technical Characteristics Affecting Left Subclavian Artery Stent Patency In Patients Managed With Fenestrated Thoracic Endovascular Repair.

Variable, n/N (%) or mean±SD or median and IQR	Compression group (N=9)	Non-compression group (N=41)	p
Underlying lesion			
Degenerative aneurysms	7 (77.8)	17 (41.4)	0.04
Dissection	2 (22.2)	8 (19.5)	0.85
Penetrating aortic ulcer	0 (0.0)	8 (19.5)	0.46
Pseudoaneurysm	0 (0.0)	8 (19.5)	0.46
History of sternotomy	3 (33.3)	4 (9.7)	0.06
Arch type			
Type I	3 (33.3)	5 (12.2)	0.11
Type II	2 (22.2)	19 (46.3)	0.18
Type III	4 (44.4)	17 (41.4)	0.86
Bovine arch	2 (22.2)	9 (22.0)	0.99
LSA anatomic details			
Distance to bone structure (mm)	11.7±8.9	23.0±7.8	0.003
Distance between LCCA-LSA	9.6±5.5	12.8±6.0	0.35
Distance between LSA**-**lesion	12.6±15.2	8.0±6.9	0.17
LSA diameter at landing zone	9.1±2.6	9.8±1.4	0.36
Δd LSA ostium-landing zone	3.6±2.4	3.5±1.6	0.84
Take-off angle (°)	52.0±39.0	62.0±15.0	0.06
Tortuosity index	1.3±0.4	1.2±0.1	0.03
Pre-operative stenosis >50%	0	0	—
Circumferential atheroma >50%	0	0	—
Circumferential thrombus >50%	0	0	—
LSA dissection	0	0	—
Stent characteristics			
Balloon-expandable stents	9 (100)	41 (100)	—
Stent diameter	10 (8–10, IQR=2)	9 (8–16, IQR=2)	0.76
Relining	1 (11.1)	8 (19.5)	0.55
Post-dilation	7 (77.8)	32 (78.0)	0.99
Balloon for post-dilation	10 (8–14, IQR=2)	12 (10–14, IQR=0)	0.14

LCCA: left common carotid artery; LSA: left subclavian artery; Δd: diameter difference.

## Discussion

Fenestrated endovascular arch repair has become a valuable solution for aortic diseases involving the aortic arch and the descending thoracic aorta, which lack a proximal landing zone distally to the LSA. Previous studies showed encouraging results with high technical success and acceptable perioperative stroke rates.^5,9,10^ This analysis showed similar clinical findings, with low early adverse event rates, when the technique was applied for the preservation of the LSA. However, LSA stent compression was identified in 18% of cases, and drove 56% of these patients into a secondary reintervention. A conservative strategy was selected in the remaining cases after discussion with the patients and presence of stenosis <70%. None of the LSA stent compressions or reinterventions had clinical consequences in terms of stroke or arm malperfusion. Baseline anatomic factors seem to affect LSA stent compression and patency.

Reinterventions after f-Arch mainly consist of distal extensions to achieve full exclusion of the underlying disease while unplanned secondary interventions represent 10% of reinterventions during follow-up.^10^ In this analysis, 18% of patients needed a secondary intervention to preserve LSA stent patency, after incidental diagnosis of stent compression. Previous data of branched aortic arch endovascular repair showed high target vessel patency, with stent occlusion being rather a sporadic event.^15^ When focusing on the preservation of the LSA, branched devices offer a high freedom form reintervention, estimated at 97% at 3 years of follow-up, while branch occlusion has been diagnosed in 6% of cases during the midterm follow-up.^15–17^ Previous comparative data showed that both fenestrated and branched devices have comparable outcomes in terms of target vessel occlusion.^12^ Early identification of LSA stent compression and further, reintervention before occlusion may be beneficial for clinical consequence prevention. Conservative management with close follow-up in lower grade stenosis (<70%) may also represent a safe strategy. Appropriate patient selection and discussion with the patient about the risk and benefits of each solution may provide an individualized optimal approach.

All patients in this analysis were managed using balloon-expandable covered stents, and so that, stent diameter, length, as well as the application of relining during the index procedure did not affect the findings. Data on the role of relining of supra-aortic trunks’ bridging stents are limited and showed that it is preferably performed in branches to the LCCA rather than the LSA, potentially as a measure for stroke prevention.^15^ Previous data from f-Arch devices, applied without bridging of the corresponding fenestration, showed also encouraging target vessel patency rates.^18,19^ However, the risk of endoleak in unbridged fenestrations should be acknowledged. Hemodynamic models, that studied the LSA-branched devices, showed that stent protrusion into the aortic lumen of more than 5 mm was related to potential thrombus formation.^20^ The protrusion of the bridging stent in f-Arch cases is generally limited to less than 5 mm, while the application of flaring in all cases decreases the risk of flow turbulences at the site of bridging stent and main endograft connection.

When assessing anatomic parameters, multiple factors seemed to affect LSA stent compression, including the distance from the nearest bone structure and tortuosity index. Anatomic evaluation data of supra-aortic trunks in patients managed with endovascular aortic arch repair are lacking while data from patients managed with endovascular means for acute ischemic stroke showed that steep left common carotid angle and arch type affected the technical success of the procedure.^21^ Studies focusing on computational models, in patients with or without bovine arch variant, showed that arch type III and landing to Ishimaru zones 2 and 3 were related to higher graft migration forces.^22,23^ To our knowledge, the impact of aortic arch anatomy on target vessel patency has not been further investigated yet.

Focusing on LSA anatomic characteristics, we were able to identify risk factors for LSA bridging stent-related adverse events, including patients with tortuous LSAs at low distance from bone structures. The tortuosity and angle of target vessels have been previously investigated as factors of target vessel instability in complex endovascular abdominal and thoracoabdominal aneurysm repair.^24–26^ However, similar data on endovascular arch repair are not available in the literature despite that both factors may affect stent configuration after deployment and during follow-up. Regarding the distance from the nearest bone structures as a factor of stent compression, potential forces related to the mobility of the shoulder joint, muscle contraction and absence of soft tissues between the LSA landing zone and bones may lead to stent fatigue, compression, and fracture. Knowledge of this risk factor may promote future investigation of anatomic parameters’ impact on bridging stent outcomes after endovascular aortic arch repair.

All reinterventions were performed endovascularly without clinical consequences, and despite the limited follow-up, no recurrence was recorded. Additional stenting was applied in all cases, using balloon-expandable stents. Left subclavian artery stent compression seems to be an event mostly evolving within the midterm follow-up. Among the 9 patients presenting compression in this analysis, only 1 was diagnosed within the early post-operative period. These findings signify the importance of surveillance, including adequate imaging and potential reinterventions before clinical evolvement of stent compression. Conservative management may be also applied in cases with lower grade stenosis and according to patient’s preferences. An individualized management may be beneficial in patients presenting with asymptomatic LSA stent compression.

## Limitations

The retrospective nature of the analysis as well as the limited number of cases and events introduce significant bias. A matched analysis between patients with and without LSA stent compression was not attempted as it would to significantly underpowered findings. Multicenter data could provide more light on this topic by increasing the included patients. The limited follow-up, estimated at 18 months for the total cohort, and the loss to follow-up at 35% affected the findings of this analysis, as long-term data may be able to reveal additional factors affecting LSA bridging stent patency. Despite that the distance from adjacent bone structures was related to stent compression, details on patient’s activity, including sports performance and anamnesis of work status were missing and could not be investigated, as potential factors affecting patency. In addition, undiagnosed thoracic outlet syndrome in these patients as well as the heterogeneity of the included underlying diseases may represent confounders affecting LSA stent performance. All LSA stent-compression events were subclinical, however, a decision to perform a secondary intervention was chosen in 56% of patients, not only to reduce future potential stroke and arm malperfusion but also as a measure to prevent spinal cord ischemia in cases needing extensive coverage. Further data on the role of LSA anatomy, in addition to the characteristics of bridging stents, are needed to identify risk factors affecting target vessel patency in patients managed with endovascular arch repair.

## Conclusion

Left subclavian artery stent compression in patients managed with f-Arch affected 1 in 5 cases, without major clinical consequences. Baseline anatomic parameters, including distance of the LSA from the nearest bone structure and tortuosity affected LSA stent compression. Stent characteristics did not affect outcomes.
